# IL-8 and IP-10 expression from human bronchial epithelial cells BEAS-2B are promoted by *Streptococcus pneumoniae* endopeptidase O (PepO)

**DOI:** 10.1186/s12866-017-1081-8

**Published:** 2017-08-24

**Authors:** Jiaqiong Zou, Long Zhou, Chunlan Hu, Peng Jing, Xiaolan Guo, Sulan Liu, Yan Lei, Shangyu Yang, Jiankang Deng, Hong Zhang

**Affiliations:** 1 Department of Laboratory Medicine, The Affiliated Hospital of North Sichuan Medical College, 63 Wenhua Road, Shunqing District, Nanchong, Sichuan 637000 China; 2 Department of Laboratory Medicine, North Sichuan Medical College; Translational Medicine Research Center, North Sichuan Medical College, Nanchong, Sichuan China; 3 Department of Laboratory Medicine, Chongqing Three Gorges Central Hospital, Wanzhou, Chongqing 404100 China; 4 Department of General Medicine, Chongqing Three Gorges Central Hospital, Wanzhou, Chongqing 404100 China; 5 Department of Pediatric Surgery, The Affiliated Hospital of North Sichuan Medical College, Nanchong, Sichuan China

**Keywords:** Il-8, IP-10, BEAS-2B, PepO

## Abstract

**Background:**

The bronchial epithelium serves as the first defendant line of host against respiratory inhaled pathogens, mainly through releasing chemokines (e.g. interleukin-8 (IL-8), interferon-induced protein 10 (IP-10) etc.) responsible for neutrophil or lymphocyte recruitment to promote the clearance of inhaled pathogens including *Streptococcus pneumoniae (S. pneumoniae)*. Previous studies have shown that IL-8 expression is induced by pneumococcal virulence factors (e.g. pneumolysin, peptidoglycan-polysaccharides, pneumococcal surface protein A (PspA) etc.), which contributes to the pathogenesis of pneumonia. Whether other pneumococcal virulence factors are involved in inducing chemokines expression in epithelium is still unknown.

**Results:**

We studied the effect of PepO, a widely expressed and newly discovered pneumococcal virulence protein, on the release of proinflammatory cytokines, IL-8 and IP-10, from human bronchial epithelial cell line BEAS-2B and identified the relevant signaling pathways. Incubation of BEAS-2B with PepO resulted in increased synthesis and release of IL-8 and IP-10 in a dose and time independent manner. We also detected the increased and sustained expression of TLR2 and TLR4 transcripts in BEAS-2B stimulated by PepO. PepO activation leaded to the phosphorylation of MAPKs, Akt and p65. Pharmacologic inhibitors of MAPKs, PI3K and IκB-α phosphorylation attenuated IL-8 release, while IP-10 production was just suppressed by inhibitors of IκB-α phosphorylation, PI3K and P38 MAPK.

**Conclusion:**

These results suggest that PepO enhances IL-8 and IP-10 production in BEAS-2B in a MAPKs-PI3K/Akt-p65 dependent manner, which may play critical roles in the pathogenesis of pneumonia.

## Background

The bronchial epithelium plays an important part in the pulmonary innate immune system and serves as the first defendant line against respiratory inhaled pathogens [1, 2]. After stimulation by microbial components, bronchial epithelial cells can directly initiate innate immune responses through secreting cytokines, chemokines or other mediators, which is closely linked to inflammatory processes [3, 4]. The bronchial epithelial cells’ innate immune responses are of significance for the pathogenesis of pneumonia. *S. pneumoniae* remains the major pathogen leading to community-acquired pneumonia worldwide [5]. As evaluated by World Health Organization (WHO), every year pneumococcal infections caused 476,000 deaths in children younger than 5 years old [6].

IL-8, a member of C-X-C chemokines and primarily secreted by epithelium, is a potent chemoattractant of neutrophils and involves in the pathogenesis of acute or chronic lung diseases [7, 8]. Neutrophils predominate within cellular infiltrates and play vital roles during pneumococcal pneumonia [5]. During serotype 3 pneumococci infection, treatment with anti-neutrophil antibody leaded to widespread infection and increased mortality [9]. In high dose of serotype 4 *S. pneumonia* infection, not low inoculum, depletion of neutrophils worsened infection [10]. These researches suggest a protective role for IL-8 in pneumococcal infection. Previous studies have shown that pneumococcal virulence factors (e.g. peptidoglycan-polysaccharides, pneumolysin, pneumococcal surface protein A (PspA) etc.) can induce IL-8 expression [11–14]. Whether other pneumococcal virulence factors, especially those newly discovered virulence factors, are involved in inducing IL-8 expression is still unknown.

IP-10, a classical IFN-γ-inducible CXCR3 ligand, is a multifunctional chemokine. Its physiological activity includes recruiting natural killer (NK) cells into peripheral tissue and accelerating CD4^+^ T lymphocytes migrating toward dendritic cells (DCs) to initiate acquired immune responses [15]. It is well established that NK cells and CD4^+^ T lymphocytes play vital roles in host defense against pneumococci [5, 16]. However, there are few reports on the induction of IP-10 expression by pneumococcal components. Further researches are still needed, especially for those newly discovered pneumococcal virulence factors.

PepO is a newer and multifunctional pneumococcal virulence protein. As a virulence factor, PepO promotes pneumococci adherence to host cells by interacting with complement component C1q, fibronectin, and plasminogen. What’s more, PepO also help pneumococci evade the complement attack through interacting with C4BP and C1q [17, 18]. Our earlier studies have proved that PepO activates innate immune responses of mice partially in a TLR2 and TLR4 dependent manner [19]. Moreover, we showed that PepO increases phagocytosis by macrophages through TLR2-mir-155 signaling pathway [20]. Whether this multifunctional virulence protein is engaged in inducing chemokines expression in epithelium is still unknown.

In the current study, we explored whether PepO induced the production of proinflammatory chemokines in bronchial epithelium using the well-established cell line, BEAS-2B, as an in vitro model, and IL-8, IP-10, as the dominating outcome measurement.

## Methods

### Reagents

Immobilized Glutathione Column was purchased from Thermo scientific (Rockford, USA) and Ni^2+^-charged chromatographic column was provided by GE healthcare (Buckinghamshire, United Kingdom). Polymyxin B – agarose which is used for endotoxin removal was provided by Sigma Corp. (Santa Clara,CA). Rabbit monoclonal antibodies including anti-phospho-Akt, anti-Akt, anti-phospho-MAPKs, anti-MAPKs, and anti-phospho p65 were provided by Cell Signaling Technology Corporation (Beverly, MA). Mouse monoclonal antibody anti-actin was provided by Santa Cruz Biotechnology Corporation (Santa Cruz, CA). P38 MAPK inhibitor SB203580, extracellular signal-regulated kinase inhibitor U0126, JNK inhibitor SP600125, Janus kinase inhibitor AG490, phosphatidylinositol 3-OH kinase (PI3K) inhibitor LY294002, and IκB-α phosphorylation inhibitor BAY11–7082 were provided by Cell Signaling Technology. DMSO was used to dissolve AG490, BAY117082, PD98059, and SP600125, while water was used to dissolve LY294002 and SB203580. The final concentration of DMSO was 0.1% (volume/volume) in all the cell culture experiments.

### Preparation of recombinant PepO and GST

Recombinant PepO was prepared as Agarwal previously described [17]. Briefly, pJET1.2 (Fermentas) was used to clone the amplified full-length *pepO* or *gst* gene and then pET28a (Novagen) was used for protein expression. After N-terminal His_6_ tagged pneumococcal PepO and GST were produced in *E.coli*BL21 (DE3) (Stratagene), a Ni^2+^-charged chromatographic column (GE healthcare) or an immobilized glutathione column was used to purify the protein according to the manufacture^’^s instruction. Lipopolysaccharide (LPS) in protein preparation was removed as much as possible using Polymyxin B – agarose.

### Cell studies

Human bronchial epithelial BEAS-2B cells numbered CRL-9609TM were provided by ATCC (Manassas, VA) and cultured following their suggestions to roughly 75% confluency. Before performing experiments serum-free media were used to culture BEAS-2B cells for 24-h. The supernatants of PepO-treated cells were collected and conserved at −80 °C. Alternatively, these cells were lysed for RNA or protein extraction.

### Endotoxin-free solutions

The Limulus amoebocyte lyase assay (Biowhittaker, Inc., Walkersville, MD) was used to determine the concentration of LPS in solutions and the sensitivity limit is 12 pg/ml. All cell culture medium was provided by Hyclone Company (Logan, Utah). After detection, solutions contained no detectable LPS.

### PCR analysis

The PCR primers’ sequences were listed as follows: IL-8, sense: 5′- GGTGCAGTTTTGCCAAGGAG-3′, antisense: 5′- TTCCTTGGGGTCCAGACAGA-3′; IP-10, sense: 5′- TGTCCACGTGTTGAGATCAT-3′, antisense: 5′- ACCTTTCCTTGCTAACTGCT-3′; TLR2, sense: 5′- TGCGTGGCCAGCAGGTTCAG-3′, antisense: 5′- CAGGACCCCCGTGAGCAGGA-3′; TLR4, sense: 5′- TCCCGGTGTGGCCATTGCTG-3′, antisense: 5′- TCCCGGTGTGGCCATTGCTG-3′; GAPDH, sense: 5′- GGTGAAGGTCGGAGTCAACGGA-3′, antisense: 5′- GAGGGATCTCGCTCGCTCCTGGAAGA-3′. For quantitative analysis, the mixture of cDNA, primers, and SYBR Green enzyme was amplified in a real-time PCR machine from Bio-Rad Corporation. The amplification of GAPDH was used for endogenous reference. Both a standard curve and relative delta delta Ct were used for determining quantification.

### Enzyme-linked immunosorbent assay

The specific human IL-8 ELISA kit (Millipore, Bedford, MA) and human IP-10 ELISA kit (R&D Systems, Minneapolis, MN) were used to measure the levels of IL-8 and IP-10 in the collected supernatants. This assay was performed as the manufacturers instructed, and the sensitivity was 7.8 pg/ml.

### Western blot assays

PepO-treated Cells were washed using pre chilled PBS for twice, and then lysed with appropriate amount of lysis buffer (RIPA containing protease inhibitors, phosphorylase inhibitors, and SDS loading buffer). Cell debris was collected and boiled for 10 min. After being centrifuged for several seconds, the same amount of protein (10 μg) was separated by SDS–PAGE and then transferred to a PVDF membrane (Millipore, Bedford, MA). The membrane was blocked in 5% bovine serum albumin (BSA) for 2 h at 37 °C, and then incubated with indicated antibody at 4 °C overnight. The membrane was washed three times, then incubated with corresponding secondary antibodies labeled with horseradish peroxidase at 37 °C for 1 h. An ECL chemiluminescent detection system was used to detect the antibody–antigen complexes. Quantity one software (Bio-Rad, Hercules, CA) was used to quantify protein expression.

### Immunofluorescence analysis

For immunofluorescence analysis, cells were seeded on glass coverslips. After stimulated by PepO for appointed times, cells were fixed in 4% paraformaldehyde for 15 min, washed three times, permeabilized in 0.1% Triton X-100 for 5 min, washed three times, blocked in 2% BSA for 2 h, and detected using anti-phospho- p65 antibody (1:100 dilution) at 4 °C overnight. After that, cells were washed in PBS for 3 times, and incubated with Alexa Fluor 488 -labeled secondary antibody for 1 h, followed by DAPI staining for 5 min. Nuclear translocation of p-p65 was observed with the use of a Nikon ECLIPSE 80i microscope.

### Statistical analysis

The data were indicated as mean ± SD. Student’s t-test was used to determine the differences between groups. The difference was deemed significant when *P*-values <0.05. Prism 5 statistical software (La Jolla, CA, USA) was used to perform all analyses.

## Results

### Effects of PepO on IL-8 and IP-10 expression from activated BEAS-2B

**Fig. 1:**
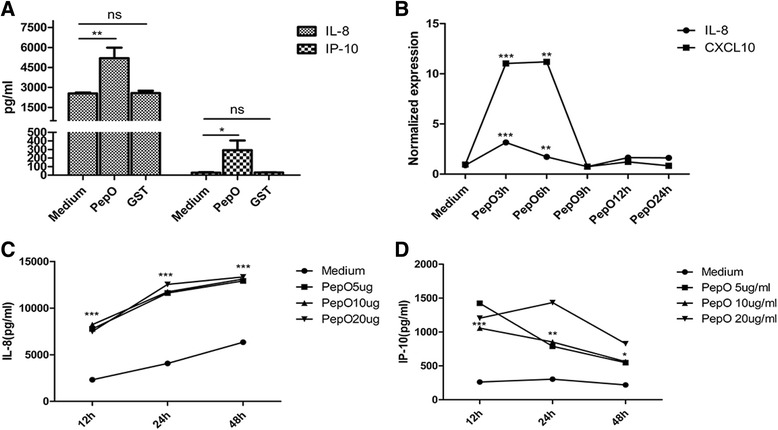
Effects of PepO on IL-8 and IP-10 synthesis and release by activated BEAS-2B. **a** After treated with medium, GST or PepO (10 μg /ml) for 24 h, IL-8 and IP-10 production in cell culture medium of BEAS-2B were determined with ELISA. **b** Quantitative RT-PCR were used to measure IL-8 and IP-10 mRNA in cells activated by PepO (10 μg /ml) for indicated times; (**c**,**d**) ELISA was used to detected IL-8 and IP-10 release in culture medium of cells stimulated by different concentration of PepO for indicated times. Results are expressed as the mean ± SD (*n* = 3). Student’s t-test was performed to calculate the statistical significance (*p < 0.05 or **p < 0.01***p < 0.001)

As shown in Fig.1a, PepO significantly up-regulated the release of IL-8 and IP-10 from BEAS-2B cells, while GST had no such effect, which demonstrated the specific effect of PepO on IL-8 and IP-10 production by BEAS-2B cells. Figure 1b revealed the kinetics response of PepO on the mRNA synthesis of IL-8 and IP-10. PepO dramatically increased the amount of IL-8 and IP-10 transcripts from BEAS-2B, showing a 3- and 11-fold increase after 3 h stimulation with 10 μg/ml PepO. We further observed that IL-8 secretion peak occurred at 48 h stimulation and IP-10 secretion peaked at 12 h in a dose independent manner (Fig. 1c, d). Therefore, in the following studies we chose 24 h as the incubation time and 10 μg /ml as the concentration of PepO.

### Effects of PepO on TLRs triggering in BEAS-2B

**Fig. 2:**
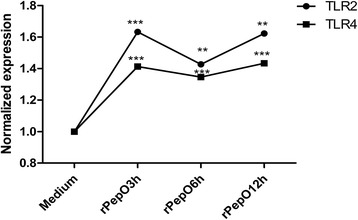
Effects of PepO on TLRs triggering in BEAS-2B. Quantitative RT-PCR were used to determine TLR2 and TLR4 mRNA in BEAS-2B activated by PepO (10 μg /ml) for indicated times; Results are expressed as the mean ± SD (*n* = 3). Student’s t-test was performed to calculate the statistical significance (**p < 0.01 or ***p < 0.001)

It is reported that inflammatory or apoptotic events can be induced by pneumococcal pneumolysin via TLR4 signaling pathway and that host innate immune responses can be elicited by glycosyl hydrolase 25 participating in invasion protein (GHIP), RrgA pneumococcal pilus type 1 protein, or adherence and virulence factor A (PavA) via TLR2 signaling pathway, which indicates that TLR2 and TLR4 mediate the recognition of pneumococcal virulence proteins [21–24]. Figure 2 shows the kinetics response of PepO-induced mRNA synthesis of TLR2 and TLR4. PepO increased the amount of TLR2 and TLR4 transcripts from BEAS-2B, showing a 1.6- and 1.4-fold increase after 3 h treatment with 10 μg/ml PepO.

### Effects of PepO on the phosphorylation of intracellular signaling molecules MAPKs, Akt, and p65 in BEAS-2B

**Fig. 3:**
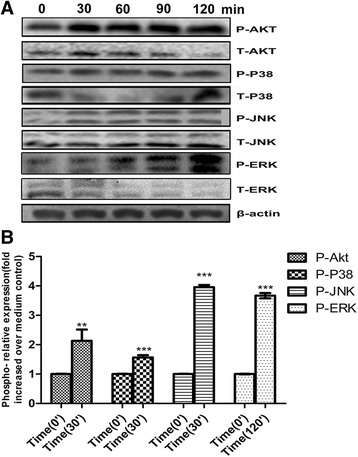
Effects of PepO on the activation of intracellular signaling molecules MAPKs and Akt in BEAS-2B. **a** BEAS-2B Cells were incubated with rPepO (10 μg /ml) for indicated times (0–120 min), lysed with lysis buffer, and the activation of p38, ERK, JNK and Akt were examined by western blot analysis. Three independent experiments with essentially identical outcome were performed, and representative blots were shown. **b** Densitometry quantification of blots from three tests were shown in histograms above. Phospho-Akt or phospho-MAPKs expression was normalized to β-actin for each sample, and they were graphed as fold change compared with medium control. Student’s t-test was performed to calculate the statistical significance (**p < 0.01 or ***p < 0.001)

**Fig. 4:**
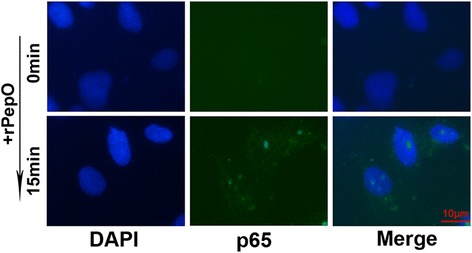
Effects of PepO on nuclear translocation of p-p65 in BEAS-2B. Nuclear translocation of p-p65 indicates the activation of NF-κB signaling pathway. Immunofluorescence assays were performed to determine the translocation of p-p65 in cells. Representative images of three independent experiments with consistent outcome were shown

As shown in Fig.3a, b, the phosphorylation of MAPKs and Akt was rapidly induced by PpeO at 30 min stimulation and sustained to 120 min. In PepO unstimulated cells p65 was seldom phosphorylated and primarily located in the cytoplasm. Fig. 4 showed that in PepO–treated BEAS-2B cells p65 was phosphorylated and translocated to their nucleus.

### Effects of pharmacological inhibitors of signaling molecules on PepO-induced IL-8 and IP-10 expression from BEAS-2B

**Fig. 5:**
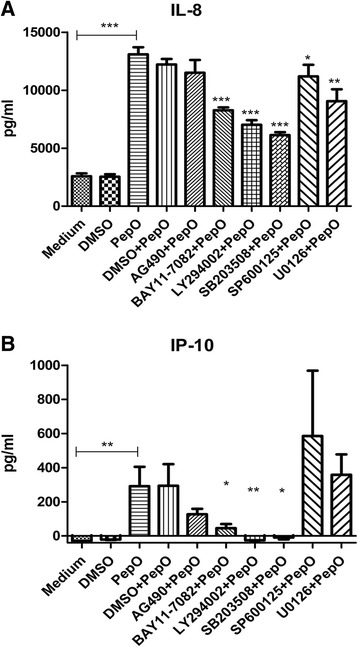
Effects of signaling molecule inhibitors on PepO-induced IL-8 and IP-10 release in BEAS-2B. **a**, **b** Cells were pre-treated with AG490 (20 μM), BAY11–7082 (10 μM), LY294002 (10 μM), SB203580 (20 μM), SP600125 (10 μM), or U0126 (20 μM) for 1 h, followed by incubation with PepO (10 μg /ml) for another 24 h. The release of IL-8 and IP-10 were determined by ELISA. DMSO (0.1%) was served as the vehicle control. Results are expressed as the mean ± SD (*n* = 3). Student’s t-test was performed to calculate the statistical significance (*p < 0.05 or **p < 0.01 or ***p < 0.001)

Figure 5a shows that PepO-induced IL-8 release was significantly suppressed by BAY11–7082, LY294002, SB203580, SP600125 and U10126, but not AG490. PepO-induced IP-10 secretion was inhibited by SB203508, BAY11–7082, and LY294002, but not AG490, SP600125, or U10126 (Fig. 5b).

## Discussion

Pneumococcal infections remain the leading reason of morbidity and mortality among children even globally, with the highest mortality occurring in Africa and Asia [6]. An array of coordinated expressed virulence factors make pneumococci succeed to colonize on the upper respiratory tract, and further invade to lower airway lumen leading to local or systematic infections [5, 25]. Identification of newer virulence factors and insights into their interaction with host could provide us with novel treatment options against pneumococcal infections.

This study aims to explore the effect of PepO, a newly discovered pneumococcal virulence protein, on the production of proinflammatory chemokines, IL-8 and IP-10, from human bronchial epithelial cells. The main finding was that PepO stimulation resulted in IL-8 and IP-10 release from BEAS-2B by a mechanism that involved in TLR2, TLR4, MAPKs, Akt and p65.

Although in pneumococcal pneumonia neutrophils predominate within infiltrated cells, the net effect of neutrophils influx to host can be good or bad [26]. Recruited neutrophils contribute to the clearance of invaded pneumococci, which is beneficial for host. Other investigators found that neutrophil depletion improved survival and decreased incidence of sepsis in mice infected with a serotype 8 strain [27], which suggested that in different models neutrophils may did harm to the host. Therefore further research is still needed to investigate the effect of PepO-induced strong production of IL-8 by BEAS-2B.

IP-10 is a potent attractant of CD4^+^ T cells and NK cells. CD8^+^ T cell recruitment can be optimized by recruited CD4^+^ T cells via producing IFN-γ to promote the local production of IP-10, which contributes to improve viral control through decreasing viral titers [28–30]. In our model, PepO-induced production of IP-10 by BEAS-2B cells may contribute to recruitment of CD4^+^ T lymphocytes and NK cells to optimize pathogen clearance. Previous study showed that early accumulation of T cells was dependent on pneumolysin [31], our results proved that PepO may also participate in T cell recruitment to airway lumen during pneumococcal infection. Actually IP-10 is a product of TLR4-TIR-domain-containing adaptor inducing IFN-β (TRIF) signaling pathway. Recent study demonstrated that TLR4-TRIF signaling pathway resulted in beneficial immunostimulatory responses in vaccine boosting [32]. Hence PepO-induced IP-10 expression by epithelium may be beneficial to the host.

The innate immune responses to pathogens generally protect host from infection. However, certain pathogens including pneumococci probably take advantage of the inflammatory response to cause invasive infection [33]. It is possible that host immune responses contribute to break the function of epithelial barrier. To reach the airway lumen, neutrophils in blood must migrate across the airway epithelium, a process during which epithelial cells reorganize its intercellular junctions to promote cells transmigration [34]. Perhaps these sites where the tight junction was reorganized can be targeted by invasive pathogens to spread to distant tissues or blood. It is suggested that pneumolysin facilitates *S. pneumoniae* passing through tissue barriers [35]. The inflammatory effect of PepO on human bronchial epithelia cell line may protect host from pneumococcal infection or contribute to invasive infection of *S. pneumoniae*. As shown by Agarwal et al., survival of PepO-mutant strain strain in whole blood was significantly decreased, indicating the vital role of PepO in pneumococcal invasion [17]. Therefore it is conceivable that pneumococci may take advantage of the inflammatory effects of PepO on bronchial epithelium.

Our experiments detected increased amount of TLR2 and TLR4 transcripts in PepO-stimulated BEAS-2B cells. A lot of microbial components can be recognized by TLR2 and TLR4. In the context of *S. pneumoniae*, it is universally accepted that TLR4 interacts with pneumolysin and TLR2 with lipid anchored membrane components [22–24]. Our previous work proved that both TLR2 and TLR4 mediate recognition of PepO by using TLR2 or TLR4 deficient mice [19]. We failed to detect TLR2 and TLR4 protein in BEAS-2B maybe because the low amount of TLR2 and TLR4 in these cells. The increased amount of TLR2 and TLR4 transcripts suggested that both TLR2 and TLR4 may participate in the effect of PepO on the expression of IL-8 and IP-10 from BEAS-2B cells.

In current study PepO-induced IL-8 and IP-10 production were regulated by different signaling pathways. ERK and JNK signaling pathways did not participate in the release of IP-10 in this model. The cross-talk between these signaling pathways is still needed to be explored.

## Conclusions

Taken together, we conclude that PepO stimulation induces IL-8 and IP-10 release from BEAS-2B cells by a mechanism dependent on MAPKs, Akt and p65 activation, and TLR2 and TLR4 may be involved in this process. The effect of PepO on bronchial epithelial cells may play critical roles in the pathogenesis of pneumonia.

## Abbreviations

BSA: Bovine serum albumin
DCs: Dendritic cells
GHIP: Glycosyl hydrolase 25 participating in invasion protein
IL-8: Interleukin-8
IP-10: Interferon-induced protein 10
NK: Natural killer
PavA: Adherence and virulence factor A
PepO: *Streptococcus pneumoniae* endopeptidase O
PspA: Pneumococcal surface protein A
*S. pneumoniae*: *Streptococcus pneumoniae*
TRIF: TIR-domain-containing adaptor inducing IFN-β
